# Endolysin LysEF-P10 shows potential as an alternative treatment strategy for multidrug-resistant *Enterococcus faecalis* infections

**DOI:** 10.1038/s41598-017-10755-7

**Published:** 2017-08-31

**Authors:** Mengjun Cheng, Yufeng Zhang, Xinwei Li, Jiaming Liang, Liyuan Hu, Pengjuan Gong, Lei Zhang, Ruopeng Cai, Hao Zhang, Jinli Ge, Yalu Ji, Zhimin Guo, Xin Feng, Changjiang Sun, Yongjun Yang, Liancheng Lei, Wenyu Han, Jingmin Gu

**Affiliations:** 1 Key Laboratory of Zoonosis Research, Ministry of Education, College of Veterinary Medicine, Jilin University, Changchun, 130062 P.R. China; 2 College of Clinical Medicine, Jilin University, Changchun, 130012 P.R. China; 3 First Hospital of Jilin University, Jilin University, Changchun, 130021 P.R. China; 4 Jiangsu Co-innovation Center for the Prevention and Control of important Animal Infectious Disease and Zoonoses, Yangzhou, 225009 P.R. China

## Abstract

Phage-derived lysins can hydrolyse bacterial cell walls and show great potential for combating Gram-positive pathogens. In this study, the potential of LysEF-P10, a new lysin derived from a isolated *Enterococcus faecalis* phage EF-P10, as an alternative treatment for multidrug-resistant *E. faecalis* infections, was studied. LysEF-P10 shares only 61% amino acid identity with its closest homologues. Four proteins were expressed: LysEF-P10, the cysteine, histidine-dependent amidohydrolase/peptidase (CHAP) domain (LysEF-P10C), the putative binding domain (LysEF-P10B), and a fusion recombination protein (LysEF-P10B-green fluorescent protein). Only LysEF-P10 showed highly efficient, broad-spectrum bactericidal activity against *E. faecalis*. Several key functional residues, including the Cys-His-Asn triplet and the calcium-binding site, were confirmed using 3D structure prediction, BLAST and mutation analys. We also found that calcium can switch LysEF-P10 between its active and inactive states and that LysEF-P10B is responsible for binding *E. faecalis* cells. A single administration of LysEF-P10 (5 μg) was sufficient to protect mice against lethal vancomycin-resistant *Enterococcus faecalis* (VREF) infection, and LysEF-P10-specific antibody did not affect its bactericidal activity or treatment effect. Moreover, LysEF-P10 reduced the number of *Enterococcus* colonies and alleviated the gut microbiota imbalance caused by VREF. These results indicate that LysEF-P10 might be an alternative treatment for multidrug-resistant *E. faecalis* infections.

## Introduction

Bacteriophages (phages) are bacterial viruses that are able to specifically infect and kill target host bacteria, resulting in the release of progeny phages^1^. Endolysins (lysins) are hydrolytic enzymes encoded by double-stranded DNA phages, and they are responsible for cleaving the cell wall peptidoglycan of target bacteria^2^. When a lysin is exogenously-added to Gram-positive bacteria, it is able to access and hydrolyse peptidoglycans and finally lyse the bacteria within seconds to minutes^3^. Bacterial cell walls are highly conserved, and they are essential for the life and reproduction of bacteria. To the best of our knowledge, there is no report on the development of bacterial resistance against lysins^4^. Recently, lysins have received considerable attention as alternative antibacterial agents due to the emergence of multidrug-resistant bacteria. In particular, lysins show great potential for combating antibiotic-resistant Gram-positive pathogens.

*Enterococcus faecalis* is a Gram-positive bacterium that is usually considered harmless and commensally colonizes the lower intestinal tract, oral cavity, and vaginal tract of humans and animals^5^. *E. faecalis* can also be found in soil, water, plants, and sewage. Despite its commensal behaviour, *E. faecalis* is also an important opportunistic pathogen. The acquisition of virulence factors and introduction of the bacterium to new areas of the body due to medical intervention have led to the designation of *E. faecalis* as a leading cause of life-threatening nosocomial infections worldwide. *E. faecalis* also causes community-acquired infections, particularly in immunocompromised patients^6–8^. It is associated with several human infections, including neonatal sepsis, peritonitis, device-related infections, infective endocarditis, wound infections, urinary tract infections, and bacteraemia^9–11^. Coupled with its intrinsic resistance to several antimicrobial agents^12^, the massive and unnecessary use of antibiotics in both human healthcare systems and animal production has led to the increased prevalence of multidrug-resistant *E. faecalis* strains^13^.

Vancomycin has been called the “last line of defence” against Gram-positive bacteria. Unfortunately, some *E. faecalis* strains have even shown resistance to vancomycin, intensifying the threat of serious infections^14^. This reduced susceptibility to antibiotics makes the treatment of *E. faecalis* infections very difficult, and therapeutic options are often very limited^15^. For this reason, there is an urgent need to develop new antibacterial agents to fight infections caused by multidrug-resistant *E. faecalis*, particularly the vancomycin-resistant strains.

Several studies have investigated the characteristics and therapeutic administration of *E. faecalis* phages. However, few studies have explored the effects of *E. faecalis* phage lysins. In particular, only Zhang *et al*. have reported on the protective effect of an *E. faecalis* phage lysin *in vivo* ^16^. Additionally, it remains unknown whether *in vivo* administration of *E. faecalis* phage lysin affects the commensal *E. faecalis* that colonizes the gastrointestinal tract. In this study, we reported LysEF-P10, a lysin that is derived from a new *E. faecalis* phage and shares only 61% amino acid identity with its closest homologues. LysEF-P10 was studied *in vitro* and *in vivo* as an alternative treatment strategy for multidrug-resistant *E. faecalis* infections.

## Results

### Genome sequence of the phage EF-P10 and analysis of its lysin

**Figure 1:**
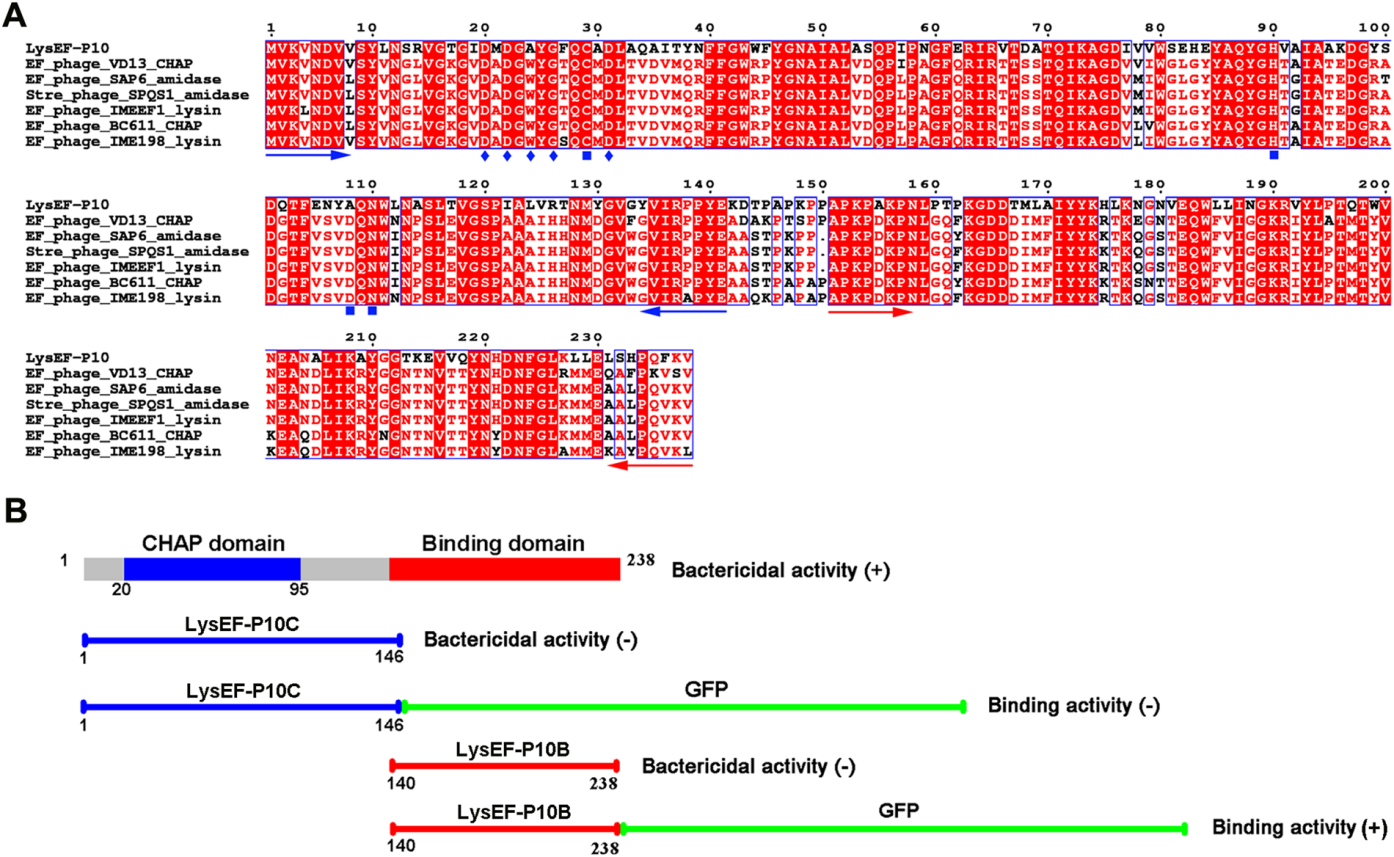
(**A**) Sequence alignment of LysEF-P10 and homologous proteins. The Cys-His-Asn triplet and putative calcium-binding residues are indicated by filled blue squares and filled blue diamonds, respectively. The blue and red arrows delineate the ends of the catalytic and binding domains, respectively. All alignments were obtained using CLUSTAL W (http://www.ch.embnet.org/software/ClustalW.html). The figure was mapped using ESPript (http://espript.ibcp.fr/ESPript/ESPript/index.php). Strictly conserved residues are boxed in white on a red background, and highly conserved residues are boxed in red on a white background. (**B**) Domain organisation of LysEF-P10. LysEF-P10 contains two domains: a CHAP domain (blue, residues 20–95) and a putative binding domain (red, residues 140–238). The figure also maps schematics of LysEF-P10C, LysEF-P10C-GFP, LysEF-P10B, and LysEF-P10B-GFP. The lytic or binding activity of these proteins is indicated with +/−.

*E. faecalis* phage EF-P10 was isolated from sewage. The genome sequence indicated that its genome is a 57,408-bp contiguous sequence of linear double-stranded DNA (Figure S1). The complete genome sequence of EF-P10 is available in GenBank under accession number KY472224. The whole genome encodes 127 putative open reading frames (ORFs). Of these ORFs, ORF 60 shows ≤61% homology with several putative lysins, including lysins derived from the *E. faecalis* phages VD13, SAP6, and IME-EFm1 and the *Streptococcus* phage SPQS1, as shown in Fig. 1(A). ORF60 consists of 238 amino acids (approximately 26 kDa) and may encode the putative lysin protein of EF-P10, LysEF-P10.

### Bactericidal activity of LysEF-P10

A search for putative conserved domains using the Position-Specific Iterated Basic Local Alignment Search Tool (PSI-BLAST) revealed that LysEF-P10 contains a cysteine, histidine-dependent amidohydrolase/peptidase (CHAP) domain (constructed as residues 20–95). Additionally, the C-terminal (residues 164–213) of LysEF-P10 shows 18% identity with the binding domain of the *Staphylococcus capitis* EPK1 peptidoglycan hydrolase, ALE-1^17^. According to this information, we designed constructs of LysEF-P10 and of the two independent domains, as shown in Fig. 1(B). LysEF-P10, LysEF-P10C (the single CHAP domain), and LysEF-P10B (the putative binding domain) were expressed and purified as soluble recombinant proteins in *Escherichia coli* (Figure S2).

**Figure 2:**
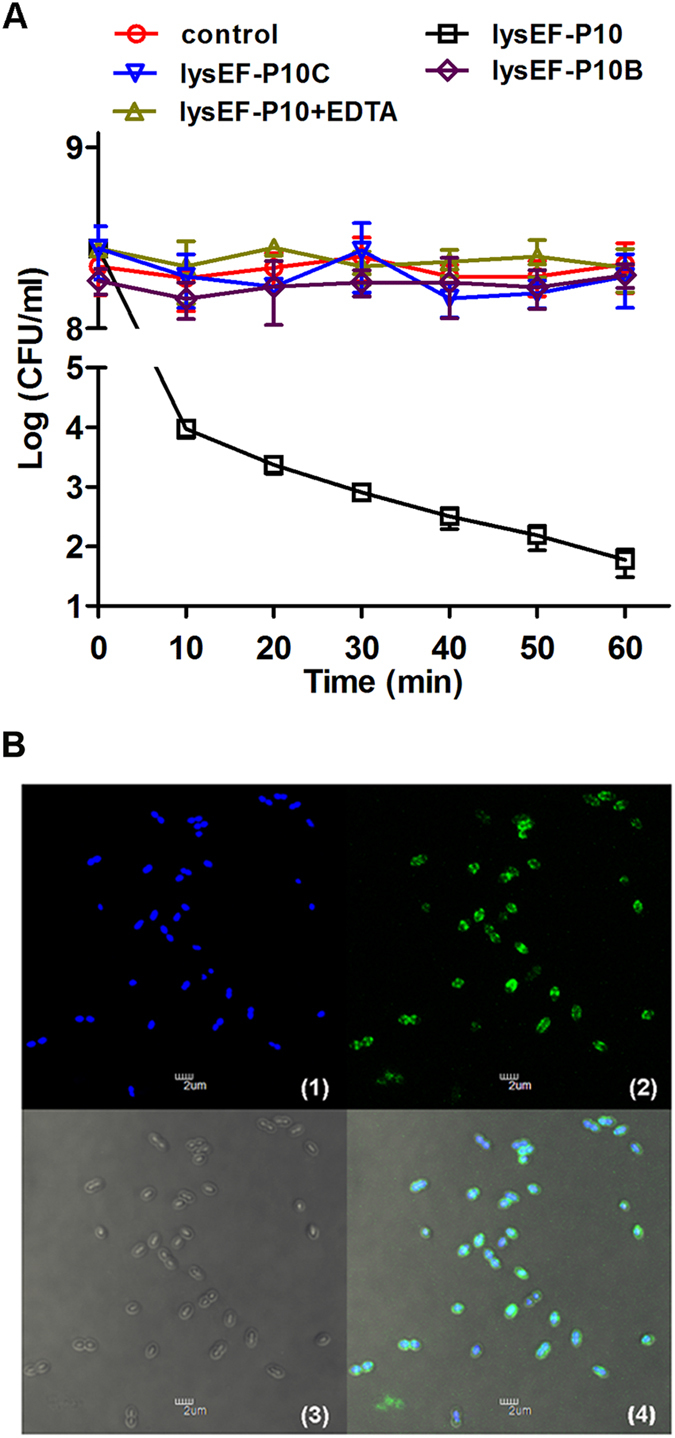
Activity of recombinant LysEF-P10 and its two domains. **(A**) Bactericidal activity. Log(CFU/ml) decrease in the *E. faecalis* N10 culture (10^8^ CFU/ml) was used to evaluate the bactericidal activity of native LysEF-P10 (20 µg/ml), LysEF-P10C (100 µg/ml), LysEF-P10B (100 µg/ml), and LysEF-P10 (20 µg/ml) pre-treated with EDTA (100 mM). As a control, N10 was treated with an equivalent quantity of Tris-HCl buffer. Error bars = ±SDs (n = 3). **(B**) Binding activity of LysEF-P10B-GFP. *E. faecalis* N10 was dyed with 20 μM/l Hoechst No. 33342 at 37 °C for 10 min and incubated with LysEF-P10B-GFP at 37 °C for 10 min. (1) Localization at 405 nm (blue, emitted by Hoechst No. 33342). (2) Localization at 488 nm (green, emitted by GFP). (3) Image with normal light. (4) Overlay of (1), (2), and (3). The bars indicate 2 μm.

**Figure 3:**
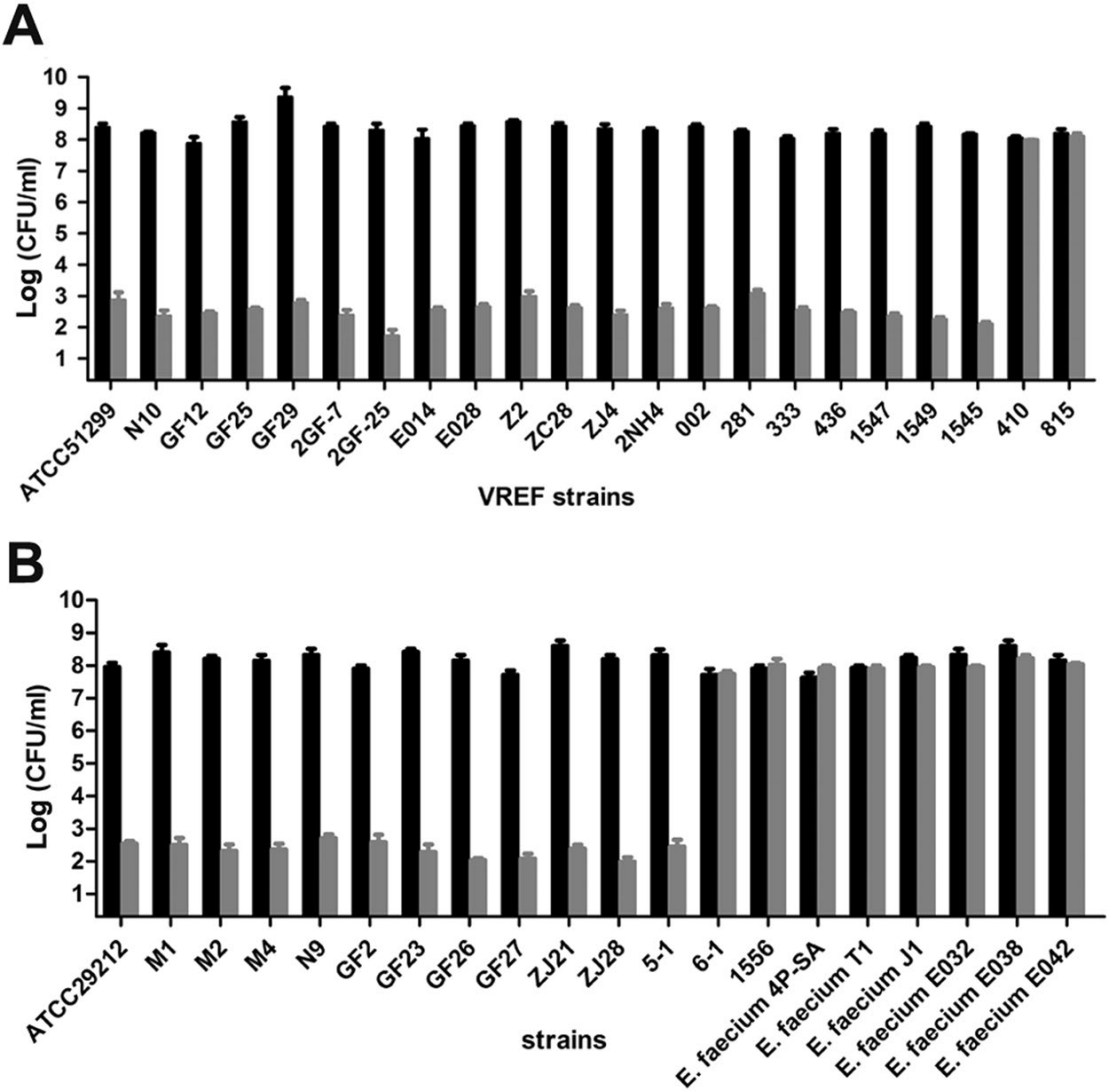
Bactericidal range of LysEF-P10 based on assays of various strains of *E. faecalis* and *E. faecium*. Log-phase cultures of different strains were exposed to LysEF-P10 (final concentration, 20 µg/ml) (grey) or buffer (black) for 1 h. The number of viable bacterial cells after treatment indicates the bactericidal activity. **(A**) VREF (*E. faecalis*) strains. (**B**) VSEF (*E. faecalis*) strains and *E. faecium* strains. Error bars = ±SDs (n = 3).

LysEF-P10 showed efficient bactericidal activity against *E. faecalis*, as shown in Fig. 2(A). After the first 10 min, viable cell numbers decreased by approximately 4 log units (and by approximately 7 log units after 60 min). LysEF-P10 was also able to kill 32/36 of a panel of diverse *E. faecalis* isolates^18^, including 20/22 VREF, as shown in Fig. 3. However, no bactericidal activity against *E. faecium* was detected. In contrast, the individual LysEF-P10C and LysEF-P10B domains, even at a higher final concentration (100 μg/ml), exhibited no bactericidal activity, as shown in Fig. 2(A).

### LysEF-P10B exhibited binding activity with *E. faecalis*

To assess the binding activity of LysEF-P10B, a fusion protein of LysEF-P10B and green fluorescent protein (GFP), designated LysEF-P10B-GFP, was expressed and purified as a soluble protein in *E. coli* (Figure S3). The green fluorescence emitted by GFP at 488 nm was only observed for *E. faecalis* N10 cells treated with LysEF-P10B-GFP, as shown in Fig. 2(B), while no green fluorescence was observed for *E. faecalis* N10 cells treated with either LysEF-P10C-GFP or GFP under the same conditions, as shown in Figure S4(A,B). Interestingly, the panel 2 of Fig. 2(B) indicates that LysEF-P10B-GFP does no bind evenly to the cell wall, but locates mainly at the midcell and two poles of *E. faecalis* cells. Additionally, no green fluorescence was observed for *E. faecium* 4P-SA cells treated with LysEF-P10B-GFP, as shown in Figure S4(C). This indicates that LysEF-P10B contains the binding domain, and the absence of bactericidal activity exhibited by LysEF-P10C may be due to the deletion of the cell wall-binding domain.

### Key amino acid analysis and bactericidal activity of LysEF-P10 mutants

**Figure 4:**
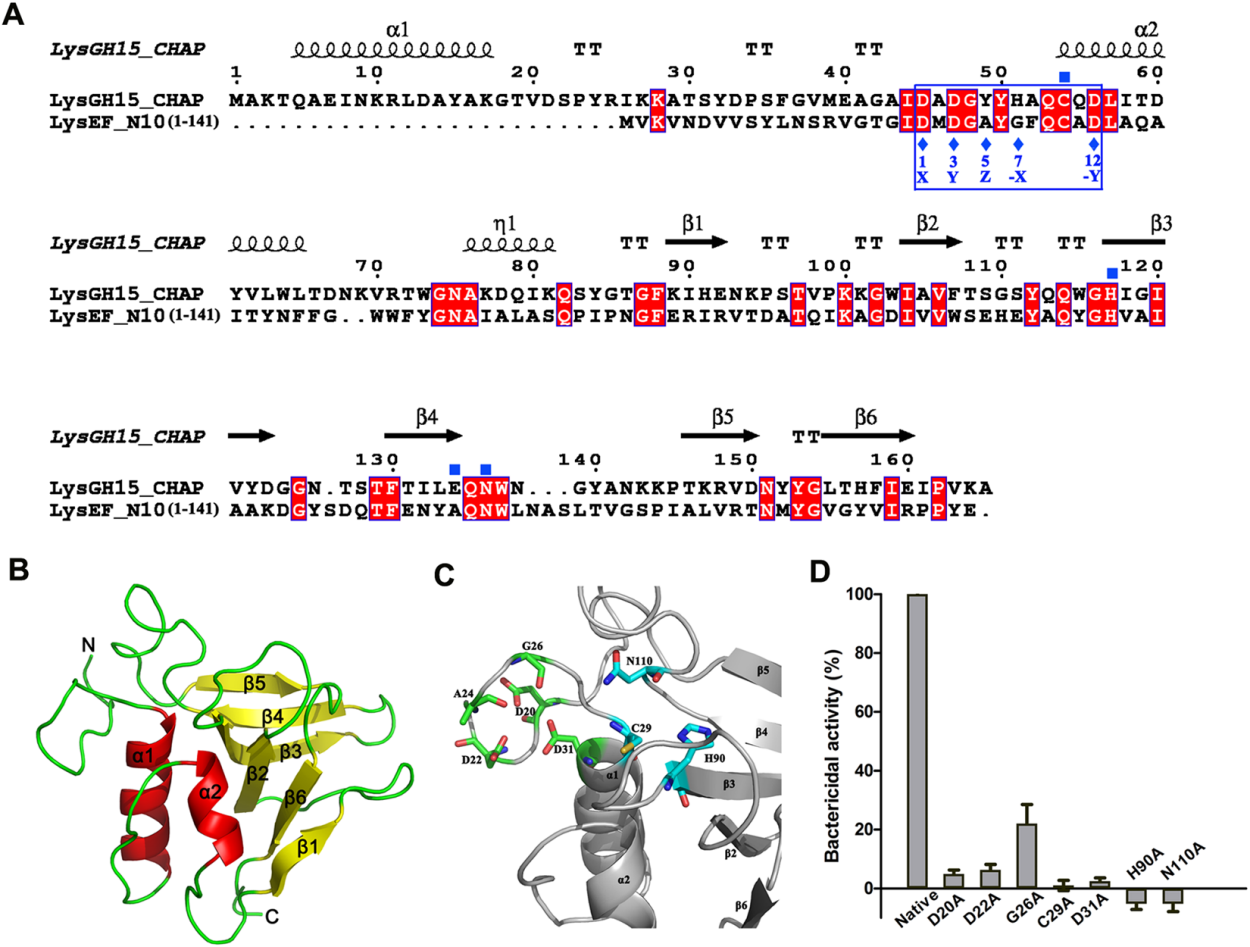
Structure model of LysEF-P10C and key residues. (**A**) Sequence alignment of the LysEF-P10C domain with the LysGH15 CHAP domain. The Cys-His-Glu-Asn quartets are indicated by filled blue squares. The 12-residue calcium-binding sites are indicated by a blue box, and positions 1, 3, 5, 7, and 12 (filled blue diamonds) are indicated by X, Y, Z, –X, and –Y, respectively. Schematic representations of the corresponding secondary structural elements are shown above the sequences. The alignment was generated using CLUSTAL W (http://www.ch.embnet.org/software/ClustalW.html). The figure was generated using ESPript (http://espript.ibcp.fr/ESPript/ESPript/index.php). **(B**) Structure model of the LysEF-P10C domain. The 3D structure model of the LysEF-P10C domain was created using Phyre2 (http://www.sbg.bio.ic.ac.uk/phyre2). **(C**) Detailed view of the putative catalytic and calcium-binding sites of LysEF-P10C. Cyan indicates the Cys-His-Asn triplet; green indicates the calcium-binding site. **(D**) Bactericidal activity of native LysEF-P10 and various mutants. The concentration of each protein was 20 µg/ml, and *E. faecalis* N10 was adjusted to 10^8^ CFU/ml. Values represent means ± SDs (n = 3).

A BLAST analysis against the Protein Data Bank (PDB) revealed that 53% of LysEF-P10 (residues 14–141, including the LysEF-P10C domain) shared the highest identity (34%, 42/125 amino acids) with the CHAP domain of *Staphylococcus aureus* phage lysin LysGH15 (PDB ID: 4OLK)^19^, as shown in Fig. 4(A). Furthermore, a 3D model of the LysEF-P10C domain was predicted using Phyre2 software with 100% confidence, using the 3D structure of the LysGH15 CHAP domain as a template, as shown in Fig. 4(B).

Based on the BLAST analysis and the 3D model that used the LysGH15 CHAP domain as the template, residues D20, D22, A24, G26, and D31 of LysEF-P10 may be responsible for binding calcium. In addition, C29, H90, and N110 (a Cys-His-Asn triplet) of LysEF-P10 may play an important role in the catalytic activity of LysEF-P10, as shown in Figs 1(A), 4(A,C) and S5.

The presence of calcium in LysEF-P10 was confirmed using an inductively coupled plasma–atomic emission spectrometry (ICP-AES) analysis (Table S1). The bactericidal activity of LysEF-P10 pre-treated with ethylenediaminetetraacetic acid (EDTA; 100 mM) was completely abolished, as shown in Fig. 2(A). The bactericidal activity of EDTA-inactivated LysEF-P10 could only be recovered when calcium was supplied, as shown in Figure S6.

To further confirm the key residues responsible for binding the Ca^2+^, several residues, including D20, D22, G26, and D31, were individually mutated to alanine. Circular dichroism (CD) spectroscopy (subtracted for buffers) showed that the mutations did not affect the secondary structures of the LysEF-P10 mutants, as shown in Figure S7. The unique calcium spectrometry signal was not detected for the D20A, D22A, or D31A LysEF-P10 mutants (Table S1). Moreover, bactericidal assays indicated that the D20A, D22A, and D31A mutations all resulted in a complete loss of bactericidal activity, as shown in Fig. 4(D). Furthermore, supplementation with calcium did not restore the activity of these three mutants (Figure S6). In contrast, the G26A was able to bind calcium and demonstrated bactericidal activity, but this activity was much weaker (approximately 25% of the activity of native LysEF-P10). We also found that all the C29A, H90A, and N110A mutations of LysEF-P10 resulted in a complete loss of bactericidal activity, as shown in Fig. 4(D).

### Elimination of VREF by LysEF-P10 in mouse models

**Figure 5:**
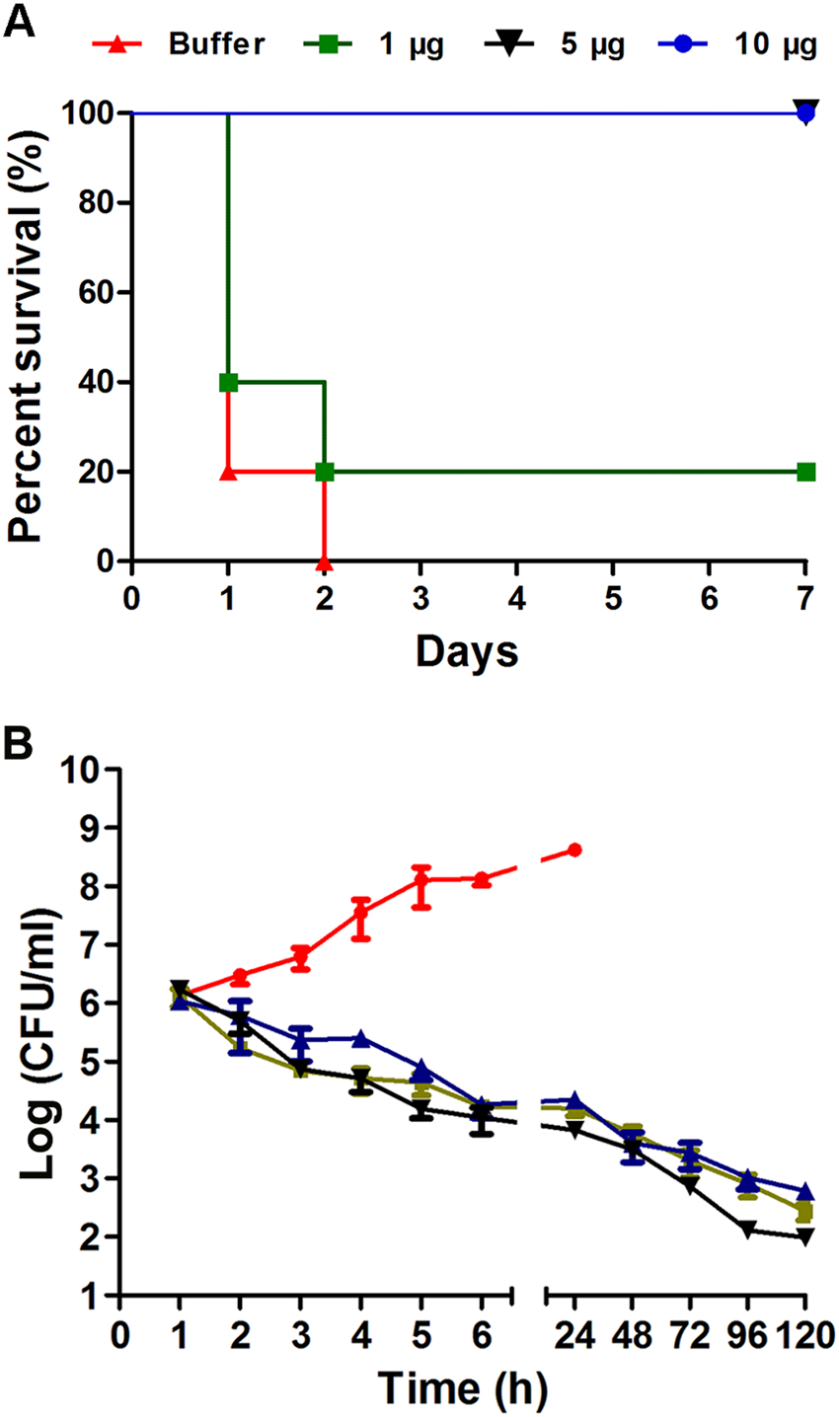
LysEF-P10 rescued mice from lethal VREF infection. (**A**) Survival rates. Mice were injected intraperitoneally (i.p.) with 4 × 10^9^ CFU of the VREF *E. faecalis* E028 strain. One hour later, various doses of LysEF-P10 were administered i.p. to treat the VREF-challenged mice (n = 10). As a control, mice were treated with an equivalent quantity of Tris-HCl buffer. (**B**) Colony counts. LysEF-P10 or an equivalent quantity of Tris-HCl buffer was administered i.p. to the mice at 1 h after the 4 × 10^9^ CFU VREF *E. faecalis* E028 challenge. At the indicated times, the bacterial counts in the peripheral blood were determined. Black line: VREF-challenged mice treated with 5 µg LysEF-P10; red line: VREF-challenged mice treated with buffer; green line: mice pre-treated with 5 µg LysEF-P10, challenged with VREF, and treated with 5 µg LysEF-P10 1 h after challenge; blue line: mice thrice immunized with 50 µg LysEF-P10, challenged with VREF, and treated with 5 µg LysEF-P10 1 h after challenge. Values represent means ± SDs (n = 3).

Intraperitoneal (i.p.) injection of ≥2 × 10^9^ colony-forming units (CFU)/mouse of the VREF *E. faecalis* E028 was sufficient to lead to a 100% mortality rate within 2 d, as shown in Fig. 5(A). In contrast, i.p. injection of 2 × 10^11^ CFU/mouse of the VREF strain N10 was not able to lead to a 100% mortality rate (data not show). Thus, VREF *E. faecalis* E028 was used for the subsequent *in vivo* studies. A load of 4 × 10^9^ cells of VREF *E. faecalis* E028 was used as the challenge dose. A high bacterial load (10^6^–10^7^ CFU/ml) was normally achieved in the blood within 1 h after challenge, as shown in Fig. 5(B). When ≥5 μg LysEF-P10 was administered, all the mice recovered (Fig. 5(A)) and bacteraemia was greatly decreased (Fig. 5(B)). The mice treated with LysEF-P10 (5 μg/mouse) had a decrease in bacterial load in the blood of approximately 3.8 log units at 24 h after treatment, as shown in Fig. 5(B). In contrast, after treatment with a buffer, the bacterial loads in the blood reached 8.3 log units at 24 h.

### LysEF-P10 activity was not affected by specific antibodies

**Figure 6:**
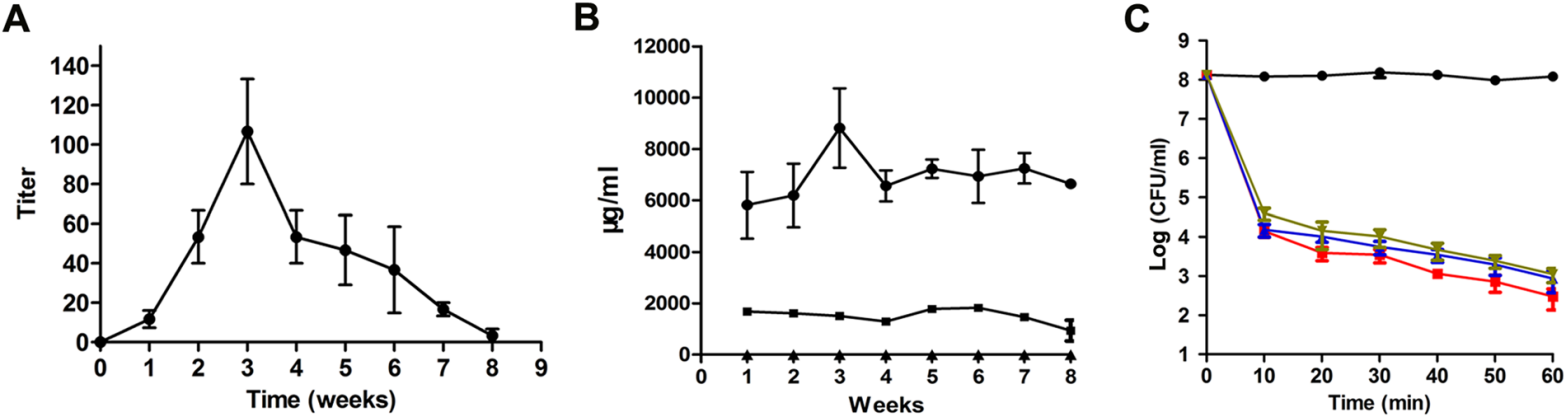
Assessment of LysEF-P10-specific antibodies. **(A**) Titres of total antibodies. Serum samples from LysEF-P10-treated (5 µg) mice were collected every week for 8 weeks. The concentrations of total antibodies were measured using ELISA. (**B**) Titres of IgG, IgM, and IgE isotypes. Concentrations of IgG (filled circles), IgM (filled squares), and IgE (filled triangles) isotypes were measured using ELISA. (**C**) Influence of anti-LysEF-P10 serum on the bactericidal activity of LysEF-P10. LysEF-P10 was pre-incubated with serum from normal mice (filled squares) or mice treated with 5 µg LysEF-P10 once (filled triangles) or immunized with 50 µg LysEF-P10 thrice (inverted filled triangles) for 10 min. The three mixtures or buffer (filled circles) alone were added to cultures of VREF *E. faecalis* E028. The log(CFU/ml) decrease in the E028 culture (10^8^ CFU/ml) was used to evaluate the bactericidal activity at various time points, as indicated. Values represent means ± SDs (n = 3).

LysEF-P10-specific antibodies were detectable at 1 week after LysEF-P10 administration and reached a peak at 3 weeks, with a 1:100 titre, as shown in Fig. 6(A). The primary antibody isotype was IgG, as shown in Fig. 6(B). The bactericidal activity of LysEF-P10 with LysEF-P10-immunized serum (1:100 titre) showed no significant difference (*P* = 0.347) compared to LysEF-P10 treated with normal mouse serum, as shown in Fig. 6(C). When 50 μg LysEF-P10 was injected subcutaneously (s.c.) three times to immunize mice, the titres of LysEF-P10-specific polyclonal antibodies in the serum collected 1 week after the final immunization were 1:32,000. This serum also did not affect the bactericidal activity of LysEF-P10 (*P* = 0.193) compared to LysEF-P10 with normal mouse serum.

We further observed that even when mice were pre-treated with LysEF-P10 (5 μg/mouse, at 21 d before injection of bacteria) or immunized with high-dose LysEF-P10 (50 μg/mouse), treatment with LysEF-P10 (5 μg) 1 h after bacterial challenge was also sufficient to protect the mice and reduce the bacteraemia, as shown in Fig. 5(B). The elimination of VREF *E. faecalis* E028 using LysEF-P10 was not affected by whether the mice were immunized with LysEF-P10.

**Figure 7:**
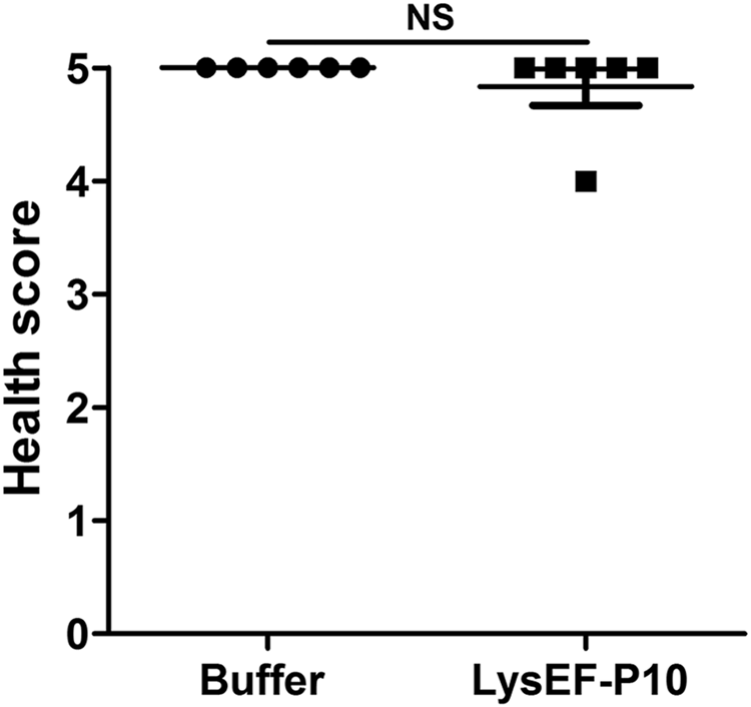
Health score. Two groups of mice (n = 6 per group) were pre-treated i.p. with 5 µg LysEF-P10. When the titre of anti-LysEF-P10 reached its peak, the mice were treated i.p. with 5 mg LysEF-P10 or an equivalent quantity of buffer. The health status of the mice was scored on a scale of 0 to 5 at 10 d after treatment. A score of 5 indicates normal health and an unremarkable condition. Slight illness was defined as decreased physical activity and ruffled fur and was scored as 4. Moderate illness was defined as lethargy and a hunched back and was scored as 3. Severe illness was defined as the aforementioned signs plus exudative accumulation around partially closed eyes and was scored as 2. A moribund state was scored as 1. Death was scored as 0. Each dot indicates the health status of a single mouse. N.S., not significant.

**Figure 8:**
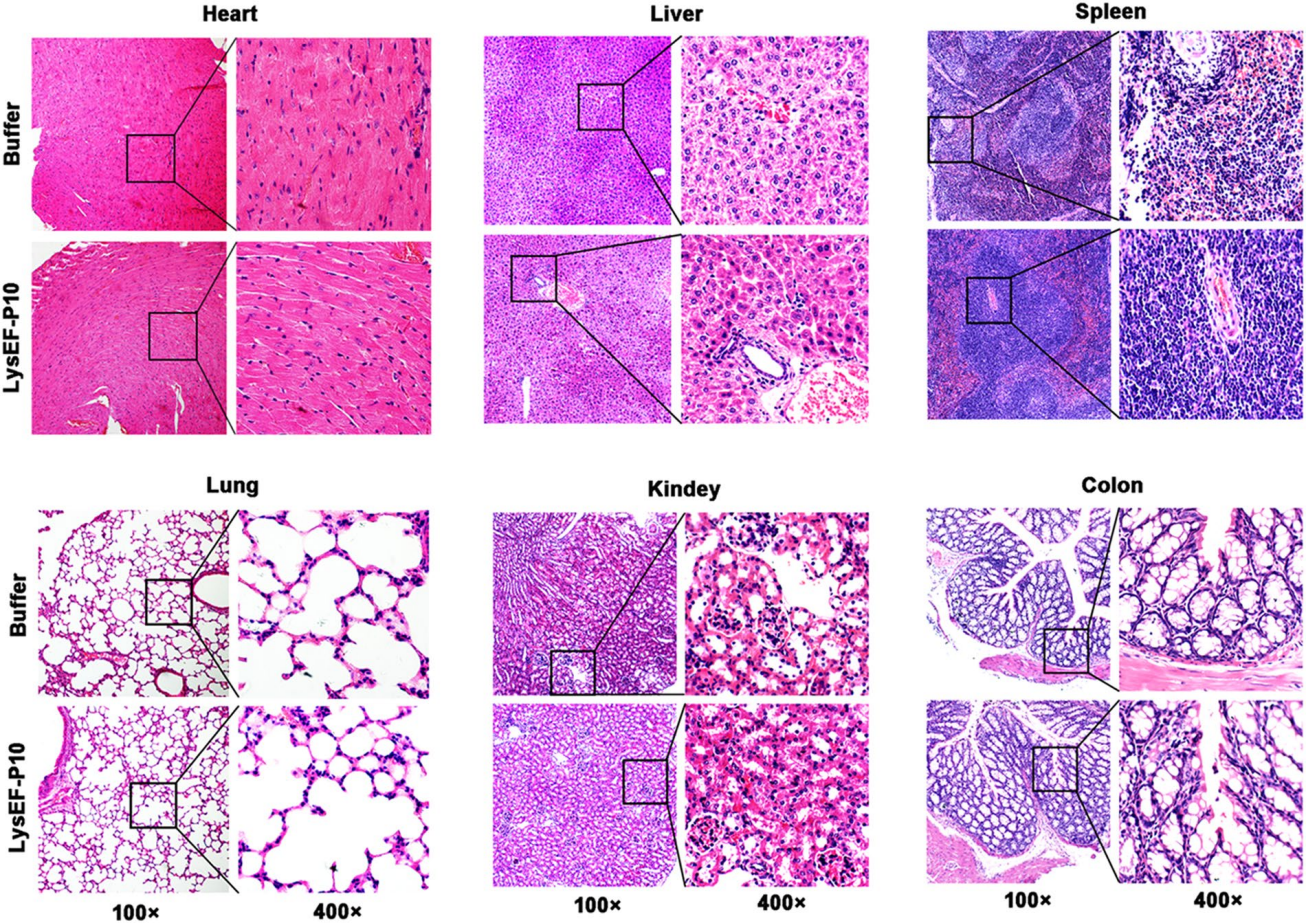
Pathological changes and organ histopathology. Two groups of mice (n = 6 per group) were pre-treated i.p. with 5 µg LysEF-P10. When the titre of anti-LysEF-P10 reached its peak, the mice were treated i.p. with 5 mg LysEF-P10 or an equivalent quantity of buffer. The heart, liver, spleen, lung, kidney, and colon were stained with haematoxylin and eosin at 10 d after treatment with 5 mg LysEF-P10 or an equivalent quantity of buffer.

Moreover, no side effects were observed when a large dose of LysEF-P10 (5 mg) was i.p. administered in the LysEF-P10-pre-treated (5 μg) mice, based on the appearance and behaviour of the treated mice, which were observed for 10 d, as shown in Fig. 7. In addition, the tissues, including heart, liver, spleen, lung, intestinal, and kidney tissues, showed no significant inflammation or other pathological changes, as shown in Fig. 8.

### Influence of LysEF-P10 on mice gut microbiota

**Figure 9:**
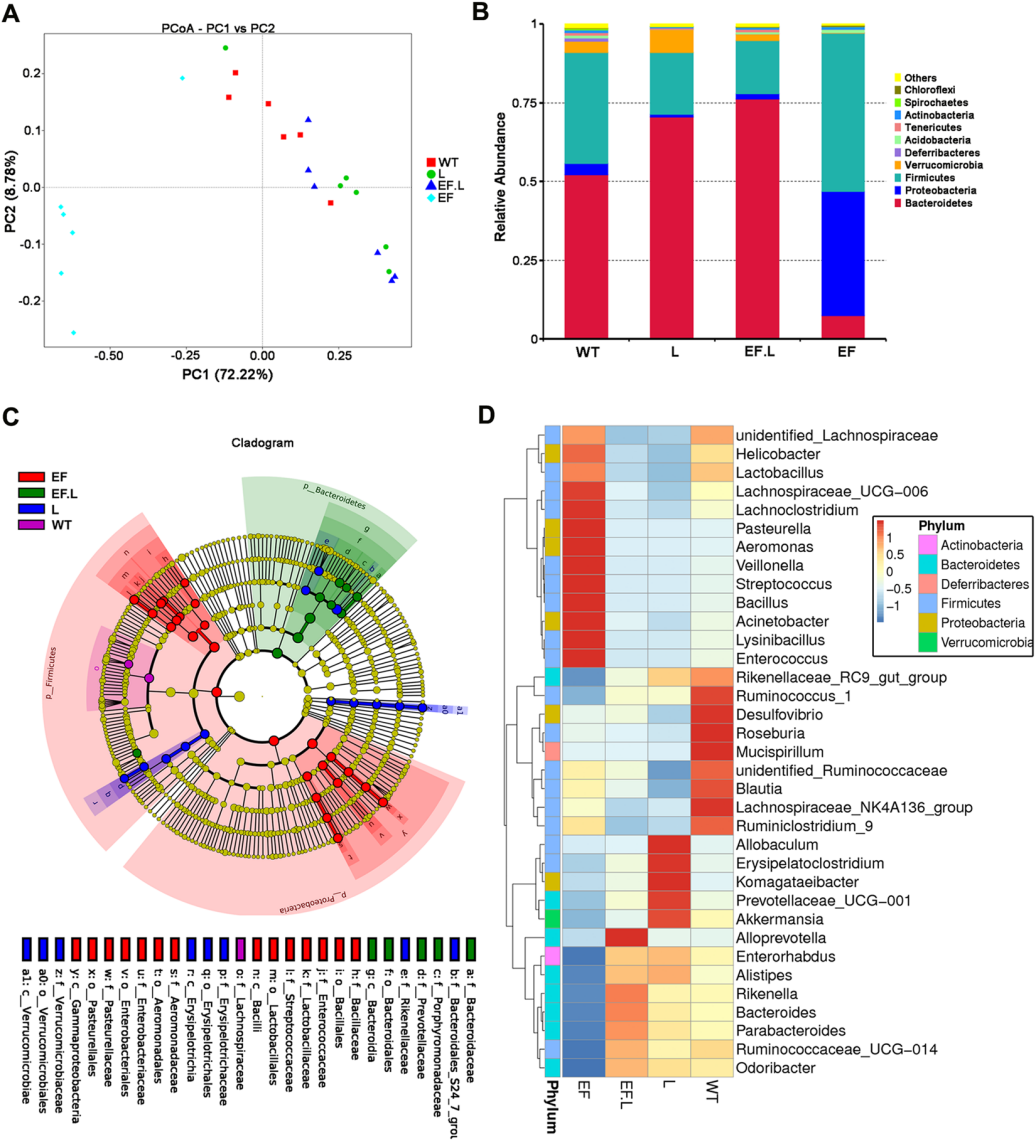
Effects of VREF challenge and LysEF-P10 treatment on gut microbiota composition. The gut microbiota composition in the faeces of VREF-challenged and -unchallenged mice treated with or without LysEF-P10 was analysed using 16S rRNA gene sequencing (n = 6 mice per group). **(A)** Plot generated using the weighted version of UniFrac-based PCoA. **(B)** Bacterial taxonomic profiling at the phylum level of identified gut microbiota. **(C)** Phylogenetic distribution of microbial lineages in the faecal samples. (**D**) Abundance of gut microbiota at the genus level. (EF): mice challenged i.p. with 4 × 10^9^ CFU/mouse of VREF *E. faecalis* E028; (EF.L): mice injected i.p. with 5 μg LysEF-P10 1 h after challenge with 4 × 10^9^ CFU/mouse of E028; (L): mice injected i.p. with 100 μg LysEF-P10 only; (WT): mice treated with buffer, as a negative control.

The intestinal microflora in the VREF-challenged group (designated the EF group), the VREF-challenged and LysEF-P10-treated group (designated the EF.L group), the LysEF-P10-treated group (designated the L group), and the group treated with buffer (designated the WT group) were analysed. After selecting effective tags, a total of 1,402,115 effective tags were generated; each faecal sample (n = 6 for each group) produced a mean of 58,421 effective tags. The mean number of operational taxonomic units (OTUs) in each sample was approximately 580. The rarefaction curve and rank abundance demonstrated that the sequencing depth covered most of the diversity (Figure S8). A UniFrac-based principal coordinates analysis (PCoA) revealed a distinct clustering of the microbiota composition for each of the four groups (Fig. 9(A)). When the mice were challenged with VREF *E. faecalis* E028 (in the EF group), the microbial composition of 5/6 mice shifted significantly compared to the WT group. In contrast, no significant shift in microbial composition, compared to that in the WT group, was observed among mice in the other two groups (the L and EF.L groups).

Taxonomic profiling demonstrated that Bacteroidetes and Firmicutes exhibited high abundance in the WT group. In contrast, the VREF challenged caused reduced Bacteroidetes, but increased Proteobacteria and Firmicutes, compared to the WT group (Figs 9(B,C) and S9). Specifically, the genus *Enterococcus* (in the family *Enterococcaceae* and the phylum Firmicutes) was enriched in the VREF-challenged group compared to the WT group (Figs 9(D) and S10). Moreover, the VREF *E. faecalis* E028 challenge changed the abundance of a variety of bacterial genera, suggesting that the balance of the gut microbiota was disrupted. However, in the LysEF-P10 treated group (5 µg), the balance of the gut microbiota recovered to a great degree. In particular, the genus *Enterococcus* in the VREF-challenged mice was reduced after treatment with LysEF-P10 (Fig. 9(D)). Again, most of the alterations in the abundance of specific genera in the VREF-challenged group were reversed after treatment with 5 µg LysEF-P10. Collectively, the gut microbiotas of VREF-challenged mice were recovered by LysEF-P10 due to suppression of the genus *Enterococcus*, resulting in a microbiota composition similar to that of normal mice. Additionally, when a large dose of LysEF-P10 (100 μg) was administered to normal mice, the abundance of the genus *Enterococcus* was significantly reduced, resulting in alteration of the microbiota composition compared with the normal group (Figs 9(D) and S10).

## Discussion

In this study, a new phage, EF-P10, was isolated using *E. faecalis* as the host strain. This phage shows an extremely narrow host range, infecting only its host strain (*E. faecalis* strain N10). Based on a bioinformatics analysis, LysEF-P10, a putative lysin of EF-P10, was identified and expressed. Compared with the very narrow infective range of the host phage, LysEF-P10 showed a much broader bactericidal range, not only killing antibiotic-sensitive *E. faecalis* strains but also lysing multidrug-resistant strains, including VREF strains. However, LysEF-P10 did not kill other bacteria, including *E. faecium*. In contrast, in addition to killing their natural target *E. faecalis*, several other reported *E. faecalis* phage lysins, such as the amidase ORF9 of phage ΦEF24C^20^, PlyV12 of *E. faecium* phage Φ1^21^, IME-EF1 lysin^16^, EFAL-1^22^, Lys168, and Lys170^23^, reported to kill the related species *E. faecium*. Moreover, EFAL-1 can also lyse some streptococcal isolates, whereas PlyV12 has the broadest lytic spectrum as it also acts against several streptococcal and staphylococcal strains.

A broad bactericidal spectrum against the target pathogenic bacterial strains is considered an essential prerequisite for therapeutic candidates^24, 25^. However, both *E. faecium* and *E. faecalis* are usually considered harmless commensal bacteria in healthy humans and animals. Lysins with multi-species bactericidal activity may also kill normal *E. faecium* in the patient when eliminating pathogenic *E. faecalis*, or vice versa. Hence, in comparison to the other lysins, LysEF-P10 shows greater potential to control infections caused by *E. faecalis* strains, including VREF strains, without overly perturbing the commensal microflora.

LysEF-P10 shares only 61% amino acid identity with its closest homologues. Interestingly, LysEF-P10C showed some identity with the CHAP domain of LysGH15, which has been characterized in previous research^19^. Although the identity is not high, the putative 3D structure of LysEF-P10C was modelled with 100% confidence using the CHAP domain of LysGH15 as a template. We found that LysEF-P10C possesses a similar classical 12-residue calcium-binding site^26^.

Mutational analysis demonstrated that the side chains of the residues D20, D22, and D31 and the main chains of A24 and G26 in LysEF-P10 are responsible for coordinating a central Ca^2+^ ion. The calcium-binding site plays an important role as a switch that modulates LysEF-P10 between its active and inactive states, similar to the switch in LysGH15. Although LysEF-P10 is the only *E. faecalis* phage lysin to have been shown to have calcium-dependent lytic activity, the lysins of phages VD13, SAP6, IME-EF1, IME198, BC611, and SPQS1 most likely share similar characteristics, according to the BLAST sequence analysis.

In addition, the CHAP domain of LysGH15 possesses an active site involving a Cys-His-Glu-Asn quartet near the calcium-binding site, an arrangement often observed in the active sites of CHAP family proteins^19^. However, we only found a Cys-His-Asn active site near the LysEF-P10 calcium-binding site. These three residues affect the lytic activity of LysEF-P10. When any residue was changed, LysEF-P10 lost its lytic activity completely. It has been reported that the sulfhydryl group of the Cys residue in the active site of CHAP family proteins acts as a nucleophile and plays a critical role in hydrolysis^27^. Thus, LysEF-P10 may possess lytic activity due to a nucleophilic mechanism involving C29 as the catalytic nucleophile. The lysins of phages VD13, SAP6, IME-EF1, IME198, BC611, and SPQS1 may share a similar catalytic mechanism, as they also possess the complete Cys-His-Glu-Asn quartet active site, based on our BLAST analysis.

Although no putative conserved binding domain was found in LysEF-P10, part of LysEF-P10B (residues 164–213 of LysEF-P10) shows 18% identity with the binding domain of the *Staphylococcus capitis* EPK1 peptidoglycan hydrolase, ALE-1^17^. Additionally, fusion protein of LysEF-P10B and GFP showed binding activity in relation to *E. faecalis*, suggesting that LysEF-P10B indeed contains a binding domain. The LysEF-P10B-deleted LysEF-P10 mutant showed no bactericidal activity, suggesting that the lytic activity and lytic range of LysEF-P10 are determined by this binding domain^28, 29^. We assume that the binding of LysEF-P10B to its cognate receptor localizes the catalytic domain to the cell wall. Once LysEF-P10C is positioned close to its target by LysEF-P10B, catalysis can begin^30^. Thus, both the binding activity of LysEF-P10B and the catalytic activity of LysEF-P10C contribute to the highly efficient bactericidal activity of LysEF-P10.

*In vivo* experiments indicated that even a small dose (5 µg) of LysEF-P10 caused an efficient protection. Among *E. faecalis* lysins, only the lytic activity of phage IME-EF1 lysin has been studied^16^. It has been reported that administering phage IME-EF1 lysin at a dose of 200 µg at 30 min after inoculation with a lethal challenge of *E. faecalis* protected 80% of the mice. Although the *E. faecalis* strain used in the challenge differed between the previous study and this study, the lethal challenge dose of *E. faecalis* was similar. Therefore, the protective efficacy of LysEF-P10 may be higher, at least based on these limited results.

As a foreign protein, LysEF-P10 induced the formation of specific antibodies when administered to mice. Nonetheless, specific anti-LysEF-P10 antibodies were unable to neutralize the bactericidal activity of LysEF-P10 *in vitro*. To our knowledge, other phage lysins, including Clys^31^, Cpl-1^32^, LysGH15^33^, MV-L^34^, and PlySs2^35^, are not significantly inactivated by immunized serum. It is likely that the antibodies either do not block the key activity sites of the binding and catalytic domains or the affinity of these lysins to their bacterial targets is higher than for antibody–lysin binding^36^. In addition, a second administration of LysEF-P10 *in vivo* also efficiently protected mice against lethal *E. faecalis* infections. Notably, LysEF-P10 enhanced IgG levels among the total serum antibodies but did not induce the production of IgM and IgE. As IgE is the primary factor induced during most allergic reactions^37, 38^, there is a low risk of allergy from repeated administration of LysEF-P10. Furthermore, histological analysis showed that neither repeated nor large-dose infusions of LysEF-P10 resulted in inflammation or mast cell activation in major organs.

*E. faecalis* is a common commensal bacterium that primarily inhabits the lower intestinal tract of humans and animals^5^. Any factor that is capable of destroying these commensal bacteria could disrupt the balance of the gut microbiota. It has been shown that administration of antibiotics significantly impacts host physiology by reducing gut microbiota diversity, bile acid metabolism, and insulin sensitivity^39^. Whether LysEF-P10 can destroy the normal *E. faecalis* that inhabits the intestines and thus disrupt the balance of the gut microbiota should be evaluated. As the flora ratio in faeces may reflect the composition of mouse gut microbiota^40, 41^, faecal samples were analysed to understand the effect of LysEF-P10 on gut microbiota. We found that when mice were challenged with a lethal VREF strain, the abundance of *Enterococcus* bacteria in the faeces increased significantly. As *E. faecalis* is the principal member of the genus *Enterococcus*, this result indicates that the *E. faecalis* strain used in the challenge may be capable of entering and colonizing the gut.

More importantly, we found that when LysEF-P10 was administered, the abundance of the genus *Enterococcus* in the faeces fell. The lysin possesses a strict host range and only kills its natural target host or closely related species^24, 25^. Thus, the small dose of LysEF-P10 most likely eliminated the VREF used in the challenge that entered and colonized the gut. Additionally, LysEF-P10 substantially recovered the abundance of most bacterial families that underwent changes in abundance due to the challenge.

We also found that a high dose of LysEF-P10 was able to significantly lower the abundance of the genus *Enterococcus* in the faeces of normal mice. In light of the broad bactericidal range of LysEF-P10, it may also kill normal *E. faecalis* strains in the gut. Although a large dose of LysEF-P10 affected the balance in the gut microbiota, the health of the mice treated with the high doses of LysEF-P10 was not significantly influenced, based on the appearance and behaviours of the mice and tissue observation. Nevertheless, the prophylactic administration of LysEF-P10 should not be encouraged. These data suggest that lysin therapy aimed at treating opportunistic pathogens such as *E. faecalis* is feasible.

## Materials and Methods

### Ethics statement

Female BALB/c mice (aged 6 to 8 weeks, weighing 18 to 20 g) were purchased from the Experimental Animal Center of Jilin University, Changchun, China. All animal experimental procedures were performed in strict accordance with the Regulations for the Administration of Affairs Concerning Experimental Animals approved by the State Council of the People’s Republic of China (1988.11.1) and with approval of the Animal Welfare and Research Ethics Committee at Jilin University. For the animal studies, mice were randomly divided into groups. The investigators were blinded to the group allocation during the experiment and/or when assessing the outcome.

### Isolation of *E. faecalis* phage

The VREF strain N10 was used as the host strain to isolate phages from sewage samples collected from the Changchun sewer system (in Jilin Province)^42^. The double-layer agar plate method was used to detect the presence of phages and purify them^43^. The concentration of phage was assessed as previously described^44^ with some modifications. Following large-scale culturing and precipitation of the phage, the phage suspension was placed at the top of a discontinuous CsCl gradient (1.45, 1.50, and 1.70 g/ml) and centrifuged at 35,000 × *g* for 3.5 h at 4 °C. The phage band was collected and dialysed.

### Sequencing and bioinformatics analysis of the phage genome

The phage genome was extracted from the purified phage preparations using a viral genome extraction kit (Omega Bio-Tek Inc., Doraville, GA, USA). Whole-genome sequencing was conducted by GENEWIZ Biotechnology Co. Ltd. (Suzhou, China) using Illumina HiSeq 2500 sequencing. The genome sequences were assembled using the SOAPdenovo package^45^. A circular map of the phage genome was created and global genome comparisons were carried out using CGView (http://wishart.biology.ualberta.ca/cgview/)^46^. Coding sequences (CDSs) and putative ORFs were predicted using BLAST and GeneMarkS^47^. The gene and deduced amino acid sequences were BLASTed using the National Center for Biotechnology Information (NCBI) network service^48^.

### Amino acid sequence analysis, expression, and purification of phage lysin

The amino acid sequence of LysEF-P10 was analysed using BLASTP tools from the NCBI network service. All alignments were carried out using CLUSTAL W (http://www.ch.embnet.org/software/C-ustalW.htmL). The figure was generated using ESPript (http://espript.ibcp.fr/ESPript/ESPript/index.php).

All the primers are listed in Table S2. The enzyme sites and homologous arms are underlined. The putative lysin gene (*LysEF-P10*) of the phage was amplified using primers lys-F and lys-R. The CHAP domain (*LysEF-P10C*, 1–438 bp) and binding domain (*LysEF-P10B*, 439–714 bp) of *LysEF-P10* were amplified with primers based on the full-length *LysEF-P10* gene. The PCR fragments were cloned into the expression vector *PET-15b*, which contains a tobacco etch virus (TEV) site downstream of 6 × His tags^49^. The fusion genes consisting of *LysEF-P10C*/*LysEF-P10B* and green fluorescent protein (*GFP*) (*LysEF-P10C-GFP* and *LysEF-P10B-GFP*) were constructed using the Seamless Assembly Cloning Kit (Clonesmarter, Houston, TX, USA) according to the instructions^50^. The expression vector *PET-15b* (digested with *XhoI* and *BamHI*) and the PCR fragments (*LysEF-P10C*/*LysEF-P10B* and *GFP*, which contained homologous arms) were mixed. Seamless Master Mix was added to the mixture of the vector and PCR fragments and incubated at 50 °C for 15 min. The correct plasmids were transformed into a competent *E. coli* strain BL21 for expression. The proteins were expressed and purified according to a previous description^19^.

### Bactericidal activity of the lysin and its individual domains

*E. faecalis* N10 was cultured to an optical density at 600 nm (OD_600_) of 0.6 in Brain Heart Infusion (BHI) broth, washed thrice with Tris-Cl buffer (pH 7.5, 200 mM NaCl and Tris 20 mM), and adjusted to a concentration of approximately 10^8^ CFU/ml. LysEF-P10 (20 μg/ml), LysEF-P10C (100 μg/ml), and LysEF-P10B (100 μg/ml) were each added individually to the bacterial suspension, and the mixture was incubated at 37 °C for 1 h. A bacteria count was performed at 10, 20, 30, 40, 50, and 60 min after incubation. As a negative control, the N10 culture was treated with an equivalent quantity of Tris-HCl buffer. Other *E. faecalis* strains^18^ and *E. faecium* strains^49^ were also used to determine the bactericidal spectrum of LysEF-P10.

### Binding activity of LysEF-P10C-GFP and LysEF-P10B-GFP with *E. faecalis*

The binding activity was detected according to previous descriptions^51^ with some modifications. A 1-ml aliquot of logarithmic growth phase *E. faecalis* culture was collected and washed thrice using Tris-HCl buffer. The cells were incubated with 20 μM/l Hoechst No. 33342 fluorescent dye at 37 °C for 10 min, washed five times with buffer and resuspended in 100 μl buffer. The cells were incubated with either LysEF-P10B-GFP or LysEF-P10C-GFP at 37 °C for 10 min. After incubation, the cells were collected by centrifugation at 8,000 × *g* for 5 min, washed five times, and resuspended in 100 µl buffer. Laser scanning confocal microscopy (LSCM) was used to assess the fluorescence of the treated cells at various excitation wavelengths. GFP was used as a control.

### 3D structure model and key amino acid analysis of LysEF-P10

The amino acid sequence of LysEF-P10 was BLASTed against other proteins available in the PDB. A 3D structure model of LysEF-P10 was generated using the bioinformatics software Phyre2 (http://www.sbg.bio.ic.ac.uk/phyre2).

The putative key amino acid sites of LysEF-P10 that are essential for its enzymatic activity were preliminarily determined according to BLAST analysis and 3D structure prediction. To further confirm the key amino acids, the putative amino acids were mutated to alanine. Mutation plasmids were obtained using a QuikChange Site-Directed Mutagenesis Kit (Stratagene, La Jolla, CA, USA). The mutants were expressed and their antibacterial activity was determined, as in the aforementioned description.

### ICP-AES analysis

The metallic signal of the protein samples was detected using CP-AES (Varian, VISTA-MPX, Palo Alto, CA, USA) at the Changchun Institute of Applied Chemistry, Chinese Academy of Sciences^52^. The ICP-AES analysis conditions were as follows: radio frequency power, 1.15 kW; plasma gas flow rate (Ar), 15 l/min; nebulizer gas flow rate (Ar), 0.75 l/min; auxiliary gas flow rate (Ar), 1.5 l/min; and viewing height, 12 mm. The analysis was repeated three times.

### CD spectroscopy

A Chirascan CD Spectrometer (Bio-Logic Co., France) was used to detect the CD spectra^53^. Freshly prepared LysEF-P10 or mutants were adjusted to a concentration of 0.2 mg/ml in phosphate-buffered saline (PBS; 137 mM NaCl, 2.7 mM KCl, 50 mM Na_2_HPO_4_, and 10 mM KH_2_PO_4_, pH 7.4). The spectra were recorded at 20 °C using a 0.1-cm path length cuvette. Each scan was obtained by recording every 1 nm with a bandwidth of 1 nm in the range from 200 to 260 nm.

### Elimination of VREF by LysEF-P10 in a mouse model

Groups of mice (n = 5) were challenged i.p. with different doses of the VREF *E. faecalis* E028 (2 × 10^7^, 2 × 10^8^, 2 × 10^9^, and 2 × 10^10^ CFU/mouse) to determine the minimal lethal dose (MLD). Once the MLD was determined, 2× MLD was used as the challenge dose.

To determine the efficient treatment dose, different doses of LysEF-P10 (1, 5, and 10 μg/mouse, with a total volume of 100 μl) were administered i.p. at 1 h after VREF *E. faecalis* E028 challenge. Each dose group contained ten mice. As a control, a group of mice were treated with an equivalent quantity of Tris-Cl buffer. The colony count in the blood of the challenged mice was detected from 10 µl peripheral blood samples obtained from the caudal veins after treatment with the various doses of LysEF-P10 or buffer.

### Assessment of specific antibodies against LysEF-P10

Mice (n = 3) were treated i.p. with 5 µg LysEF-P10 or Tris-Cl buffer. Blood samples were collected weekly for 8 weeks, centrifuged (5000 × *g* at 4 °C for 10 min) and stored at −20 °C. The titres of LysEF-P10-specific antibody were measured using an indirect enzyme-linked immunosorbent assay (ELISA) method^33^. The IgG, IgM, and IgE titres in the sera were also measured using ELISA kits (R&D Systems, Minneapolis, MN, USA).

High-titre anti-LysEF-P10 polyclonal antibodies were produced^33^. Mice (n = 3) were first immunized with a mixture of LysEF-P10 (50 µg) and complete Freund’s adjuvant by s.c. injection. A mixture of LysEF-P10 (50 µg) and incomplete Freund’s adjuvant was used to immunize the mice by s.c. injection 7 d later and this process was repeated 14 d later. The mice were euthanized and blood samples were collected 21 d after the first immunization. The titres of anti-LysEF-P10 polyclonal antibodies were determined using an indirect ELISA method^33^.

### Influence of specific antibodies on the bactericidal activity of LysEF-P10

To determine whether the antibodies could affect the bactericidal activity of LysEF-P10, a neutralization assay was performed^33, 34^. A mixture of 20 μl LysEF-P10 solution (final concentration, 200 μg/ml) with 80 μl of serum collected from either normal mice, mice treated with a single 5 μg i.p. dose of LysEF-P10 (no dilution), or mice treated with three 50 μg doses of s.c. LysEF-P10 (dilution, 1:100) was followed by incubation for 10 min at 37 °C. Subsequently, the mixture was added to a VREF *E. faecalis* E028 culture (100 μl, 2 × 10^8^ CFU/ml) and further incubated at 37 °C. The bacterial counts were detected at intervals (10, 20, 30, 40, 50, and 60 min) for each group.

### Influence of specific antibodies on the protective effect of LysEF-P10

One group of mice (n = 10) was treated with 5 μg i.p. LysEF-P10. When the anti-LysEF-P10 antibodies reached the highest titre, the mice were challenged i.p. with VREF *E. faecalis* E028 (4 × 10^9^ CFU/mouse). Another group of mice was immunized three times with 50 μg LysEF-P10 according to the aforementioned description and challenged i.p. with VREF *E. faecalis* E028 21 d after the first immunization. Subsequently, a single dose of 5 μg LysEF-P10 was administered i.p. 1 h after the bacterial challenge. At intervals, the bacterial counts were determined using 10 μl of peripheral blood samples obtained from the caudal veins of the mice.

### Toxicity assays

Mice (n = 6) treated with 5 µg LysEF-P10 were i.p. injected with 5 mg LysEF-P10 or buffer when the antibodies reached the peak value. The appearance and behaviours of the mice were examined daily for 10 d. Health scores were determined on a scale of 0 to 5^54, 55^. After 10 d, the mice were euthanized using an i.p. injection of ketamine and xylazine and used in a histopathology analysis. The organs were removed and immediately placed in 4% formalin. The formalin-fixed tissues were stained with haematoxylin and eosin and toluidine blue^56^. Subsequently, the tissues were analysed using microscopy.

### Gut microbiota analysis

Mice were randomly divided into four groups (n = 6). The first group, designated the EF group, was challenged i.p. with 4 × 10^9^ CFU/mouse of VREF *E. faecalis* E028. The second group, designated the EF.L group, was injected i.p. with 5 μg LysEF-P10 1 h after the challenge with 4 × 10^9^ CFU/mouse of VREF *E. faecalis* E028. The faeces of each mouse in the EF and EF.L groups were collected 24 h post infection. The third group, designated the L group, was injected i.p. with 100 μg LysEF-P10 only. The mice in the fourth group, designated the WT group, were treated with buffer. The faeces of each mouse in the L and WT groups were collected 23 h post injection. Once the faeces samples were collected, they were immediately frozen and stored in liquid nitrogen until DNA extraction.

For each faecal sample, total DNA was extracted using a QIAamp DNA Stool Mini Kit (Qiagen, Crawley, UK) and frozen at −80 °C prior to PCR amplification. Primers with the barcodes 515 F and 806R^57^ were used to amplify the V4 region (300 bp) in the 16S rRNA gene. All the PCR analyses were performed with Phusion@ High-Fidelity PCR Master Mix (New England Biolabs,Boston, MA, USA). The PCR products were purified with a Qiagen Gel Extraction Kit (Qiagen, Dusseldorf, Germany) and processed with a TruSeq^®^ DNA PCR-Free Sample Preparation Kit (Illumina, San Diego, CA, USA).

The index codes were added to the library, and the library was sequenced on an Illumina HiSeq 2500 platform (Illumina, San Diego, CA, USA). Paired-end reads obtained after sequencing were assigned to samples based on their unique barcode and truncated by cutting off the barcode and primer sequence. The reads were then merged (v1.2.7, http://ccb.jhu.edu/software/FLASH/)^58^. Quality filtering of the raw tags was performed to obtain high-quality clean tags^59^ using QIIME (v1.7.0, http://qiime.org/index.html)^60^. The tags were BLASTed with the Gold database (http://drive5.com/uchime/uchime_download.html) using the UCHIME algorithm (http://www.drive5.com/usearch/manual/uchime_algo.html)^61^. Finally, the chimera sequences were removed^62^. Sequence analysis was performed using Uparse (http://drive5.com/uparse/)^63^. Sequences with ≥97% identity were assigned to the same OTU. Representative sequences of each OTU were screened for further annotation. Multiple sequence alignments were performed using MUSCLE (http://www.drive5.com/muscle/)^64^. For each sample, the complexity of species diversity was analysed using the Alpha diversity of six indices, comprising the observed species, Chao1, Shannon, Simpson, abundance-based coverage estimator (ACE), and Good’s coverage. All these indices were calculated with QIIME (v1.7.0) and displayed with R software (v2.15.3). A beta diversity analysis was also used to evaluate differences between samples in terms of species complexity, using a PCoA principal component analysis (PCA), and nonmetric multidimensional scaling (NMDS).

### Data analysis

GraphPad Prism 5 (GraphPad Software, Inc., San Diego, CA, USA) was used to analyse the data obtained by ELISA. SPSS v13.0 (SPSS, Inc., Chicago, IL, USA) was used for the statistical analysis of the other experimental data using one-way analysis of variance (ANOVA). The differences between the lysin-treated and control groups were assessed using the log-rank test for survival curves. *P* < 0.05 was considered statistically significant. The error bars in the figures represent the standard deviation (SD) of the mean.

### Data availability

The authors declare that the data supporting the findings of this study are available within the article and its Supplementary Information files, or from the corresponding author on request.

## Electronic supplementary material

Supplementary Information
