# Supplementary material for: Endolysin LysEF-P10 shows potential as an alternative treatment strategy for multidrug-resistant Enterococcus faecalis infections
Source: Sci Rep. 2017 Aug 31;7:10164. doi: 10.1038/s41598-017-10755-7 (PMC5579260; doi:10.1038/s41598-017-10755-7)
Supplement: Supplementary file 1 — Supplementary Information [file 41598_2017_10755_MOESM1_ESM.pdf]

Endolysin LysEF-N10 shows potential as an alternative treatment strategy for multidrug-resistant *Enterococcus faecalis* infections

Mengjun Cheng[1], Yufeng Zhang[1], Xinwei Li[1], Jiaming Liang[2], Liyuan Hu[1], Pengjuan Gong[1], Lei Zhang[1], Ruopeng Cai[1], Hao Zhang[1], Jinli Ge[1], Yalu Ji[1], Zhimin Guo[3], Xin Feng[1], Changjiang Sun[1], Yongjun Yang[1], Liancheng Lei[1], Wenyu Han[1,4*] and Jingmin Gu[1*]

Supplementary Information:

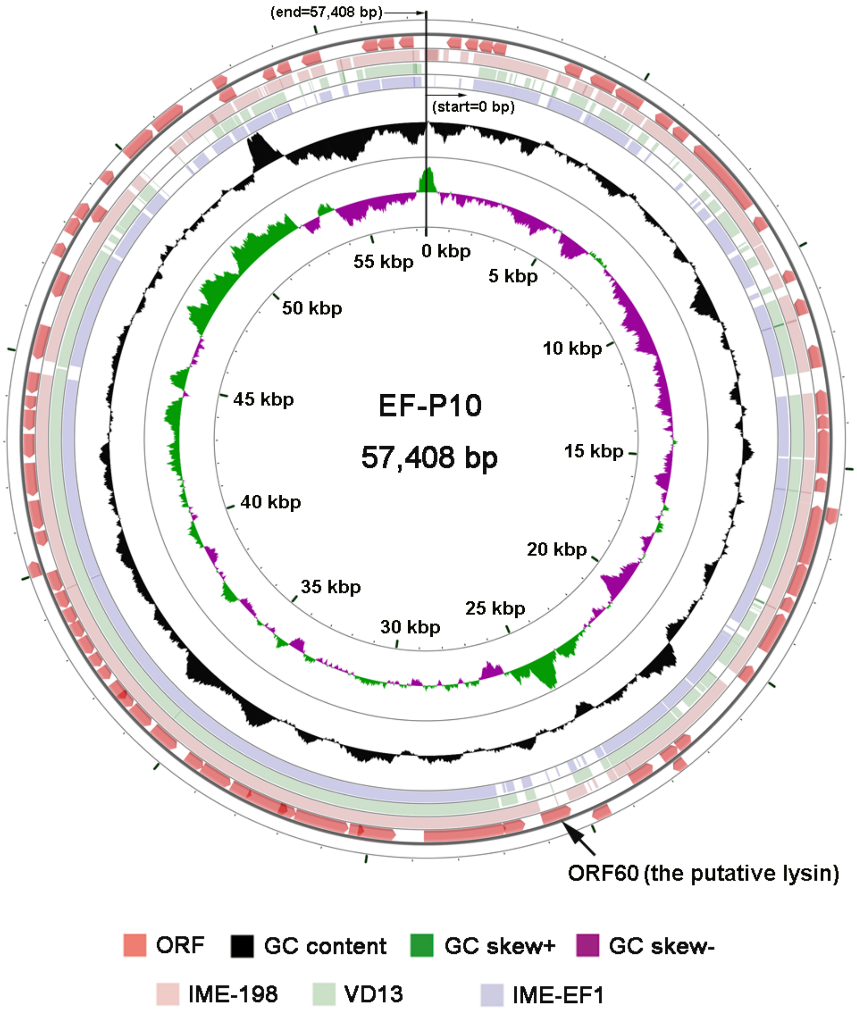

Figure S1. Genetic and physical organization of phage EF-P10 genome. The circular map of the EF-P10 genome was constructed using CGView. The genome is a contiguous sequence of linear double-stranded DNA, starting at 0 bp and ending at 57,408 bp. ORFs with >100 residues are indicated by arrows, and arrowheads point in the direction of transcription. The figure also maps GC content, GC skew, and BLASTN results with respect to phages IME-198, VD13, and IME-EF1.

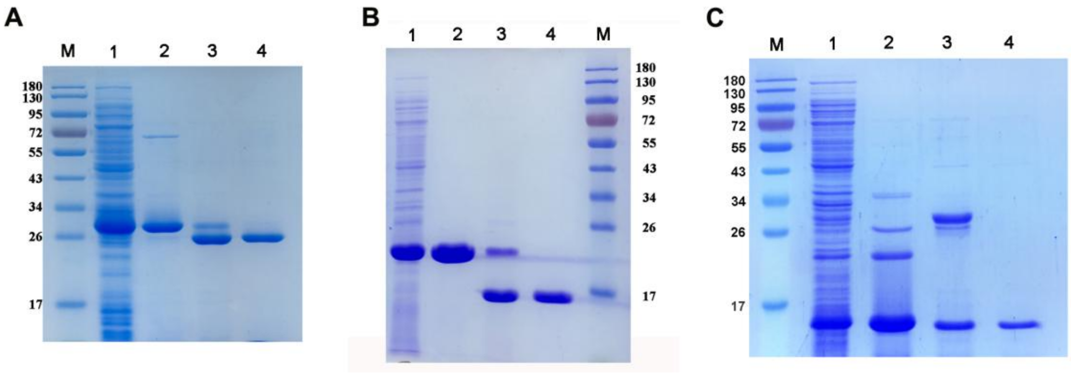

Figure S2. Protein profiles of (A) LysEF-P10, (B) LysEF-P10C and (C) LysEF-P10B. The lanes were loaded as follows: (1) crude extract of induced (1 mM IPTG at 20 ℃) *E. coli* BL21 cells containing constructed plasmid; (2) purified protein fraction eluted from Ni-NTA His•Bind slurry; (3) 6× His cut from the purified proteins using TEV protease; (4) purified protein fraction without 6× His tag; (M) molecular mass marker.

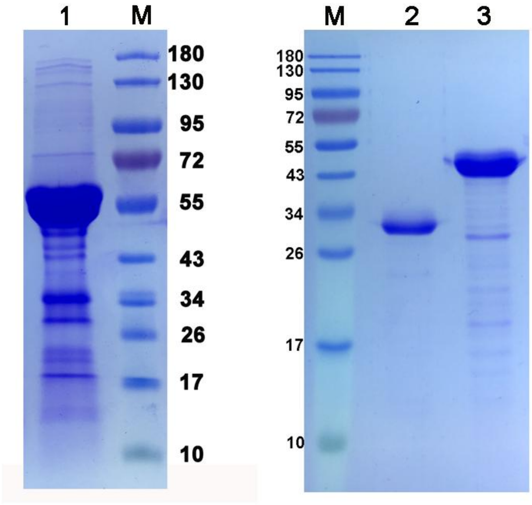

Figure S3. Protein profiles of LysEF-P10C-GFP, LysEF-P10B-GFP, and GFP. The lanes were loaded as follows: (1) purified LysEF-P10B-GFP fraction; (2) purified GFP fraction; (3) purified LysEF-P10C-GFP fraction; (M) molecular mass marker.

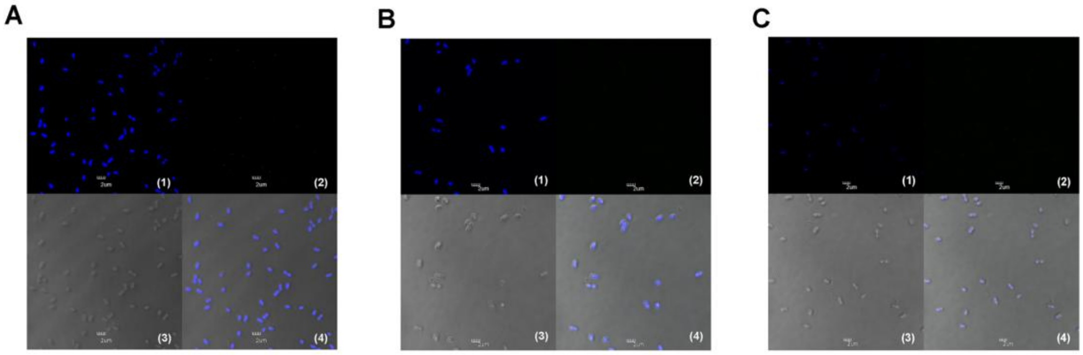

Figure S4. Binding activity of the recombinant proteins. *E. faecalis* N10 and *E. faecium* 4P-SA were dyed with 20 μM/l Hoechst No. 33342 at 37°C for 10 min. **(A)** *E. faecalis* N10 was incubated with LysEF-P10C-GFP. **(B)** *E. faecalis* N10 was incubated with GFP. **(C)** *E. faecium* 4P-SA was incubated with LysEF-P10B-GFP. (1) Localization at 405 nm (blue, emitted by Hoechst No. 33342). (2) Localization at 488 nm (green, emitted by GFP). (3) Image with normal light. (4) Overlay of (1), (2), and (3). The bars indicate 2 μm.

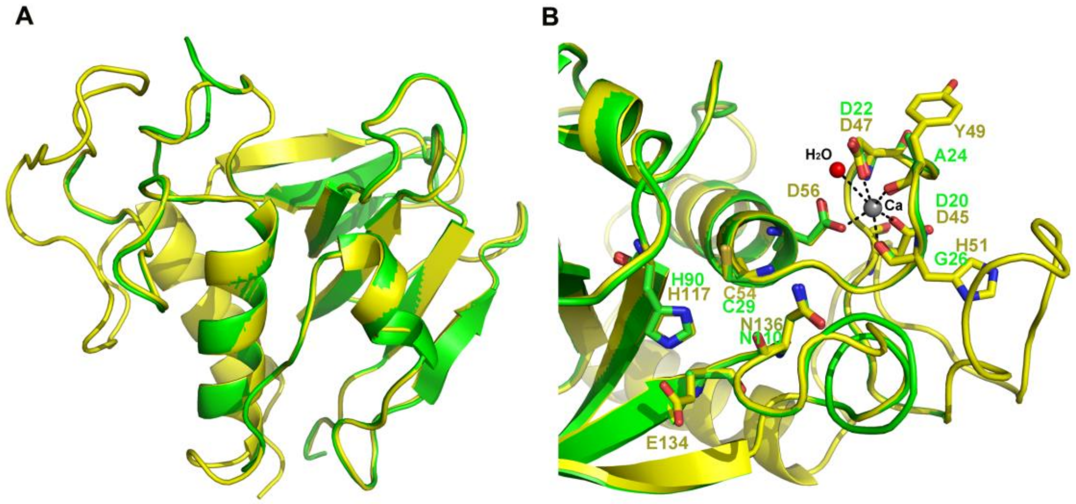

Figure S5. Structural comparison of LysEF-P10C domain (green) with LysGH15 CHAP domain (yellow). (**A**) Representation of the overall folding. Helices are shown as cylinders, and strands are shown as arrows. LysGH15 Protein Data Bank ID: 4OLK. (**B**) Detailed view of the active sites and calcium-binding sites. Calcium ions are shown as spheres.

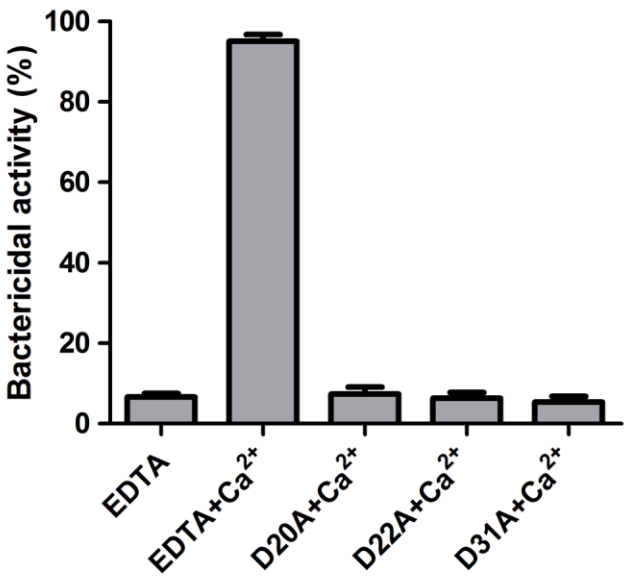

Figure S6. Bactericidal activity of LysEF-P10 and various mutants. The concentrations of proteins were 20 μg/ml, and *E. faecalis* N10 was adjusted to 10^8 CFU/ml. The concentrations of EDTA and Ca^{2+} were 100 mM and 50 mM, respectively. Values represent means ± SDs (n=3).

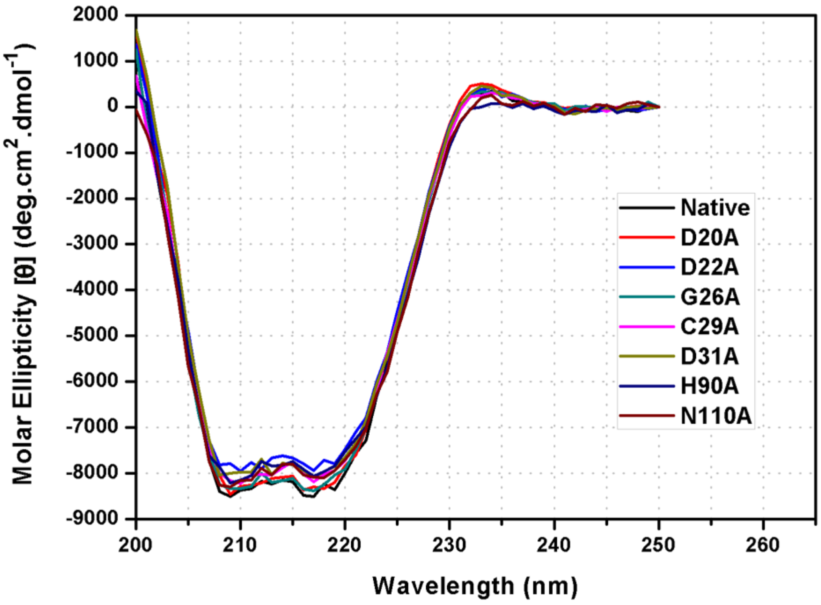

Figure S7. Circular dichroism (CD) spectroscopy of LysEF-P10 and mutants (subtracted for buffers).

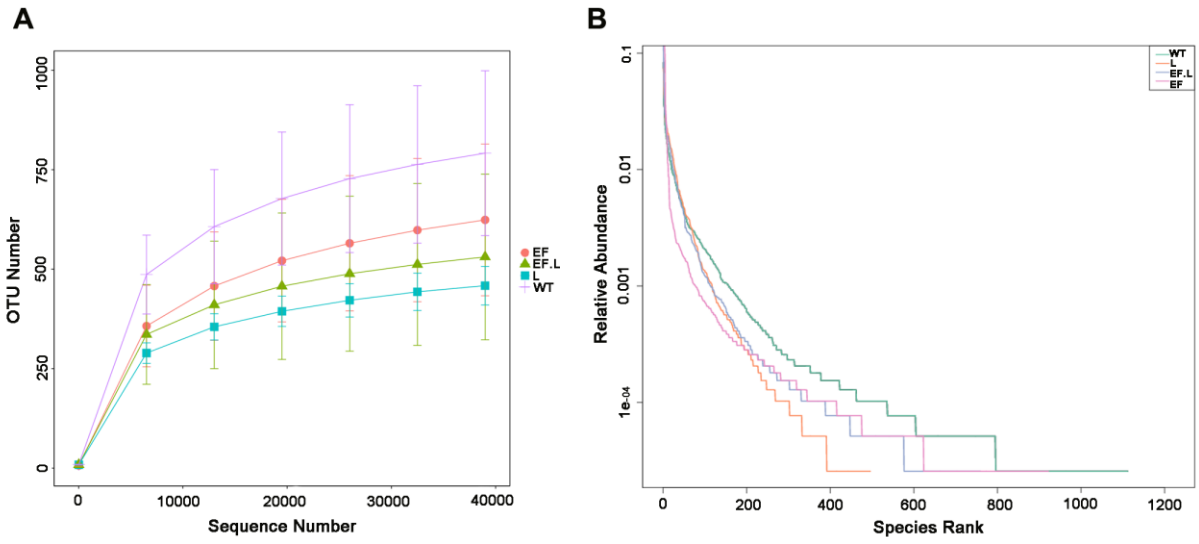

Figure S8. Alpha diversity analysis of faecal microbiota. (**A**) Rarefaction curves and (**B**) rank abundance for faecal samples obtained from normal mice treated with buffer (WT), 100 μg LysEF-P10 (L), 5 μg LysEF-P10 at 1 h after VREF challenge (EF.L), and VREF challenge alone (EF).

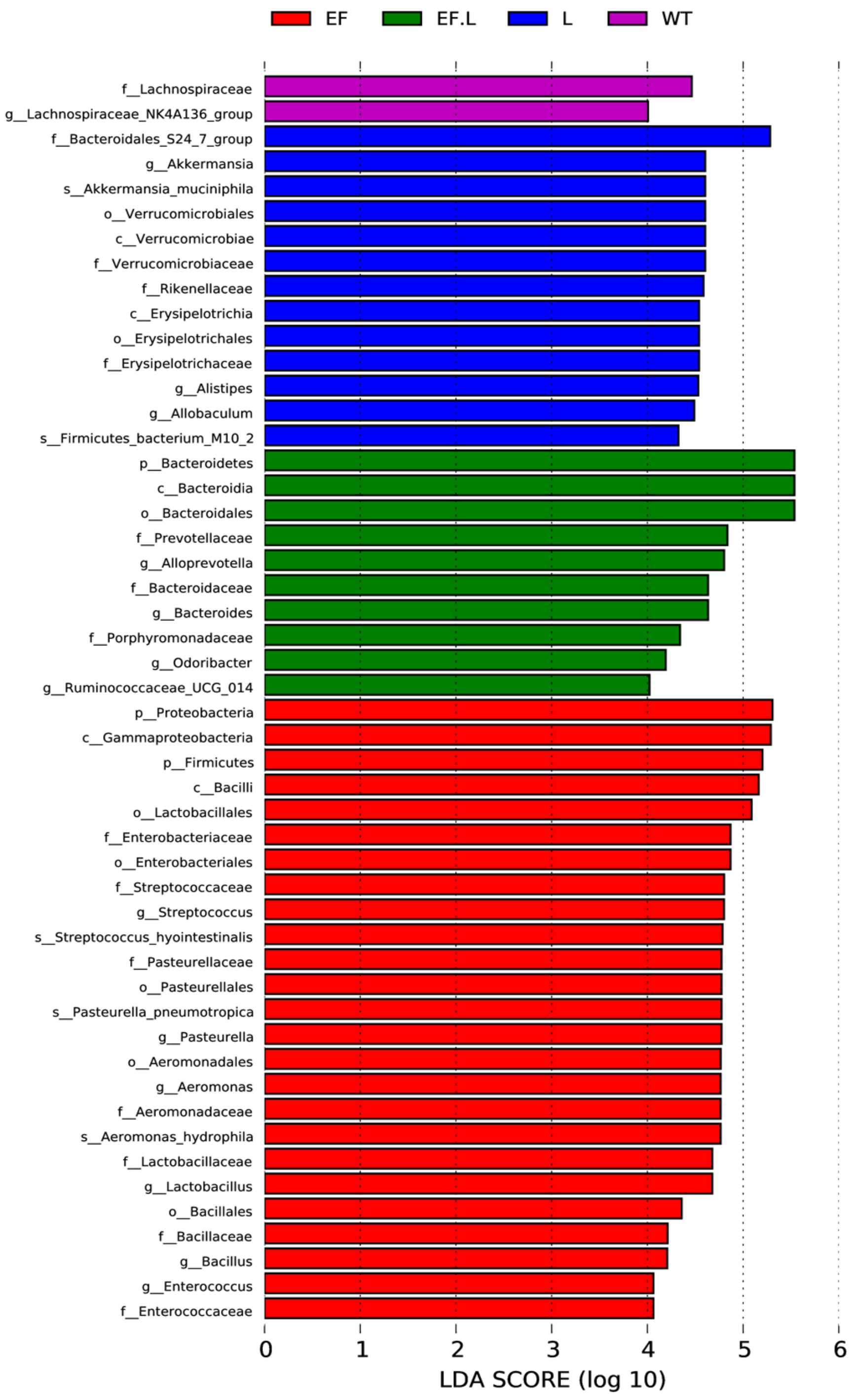

Figure S9. Overall structure and composition of gut microbiota. Differences are represented as the colour of the most abundant group. The key shows the gut microbiota phylotypes that responded to treatments.

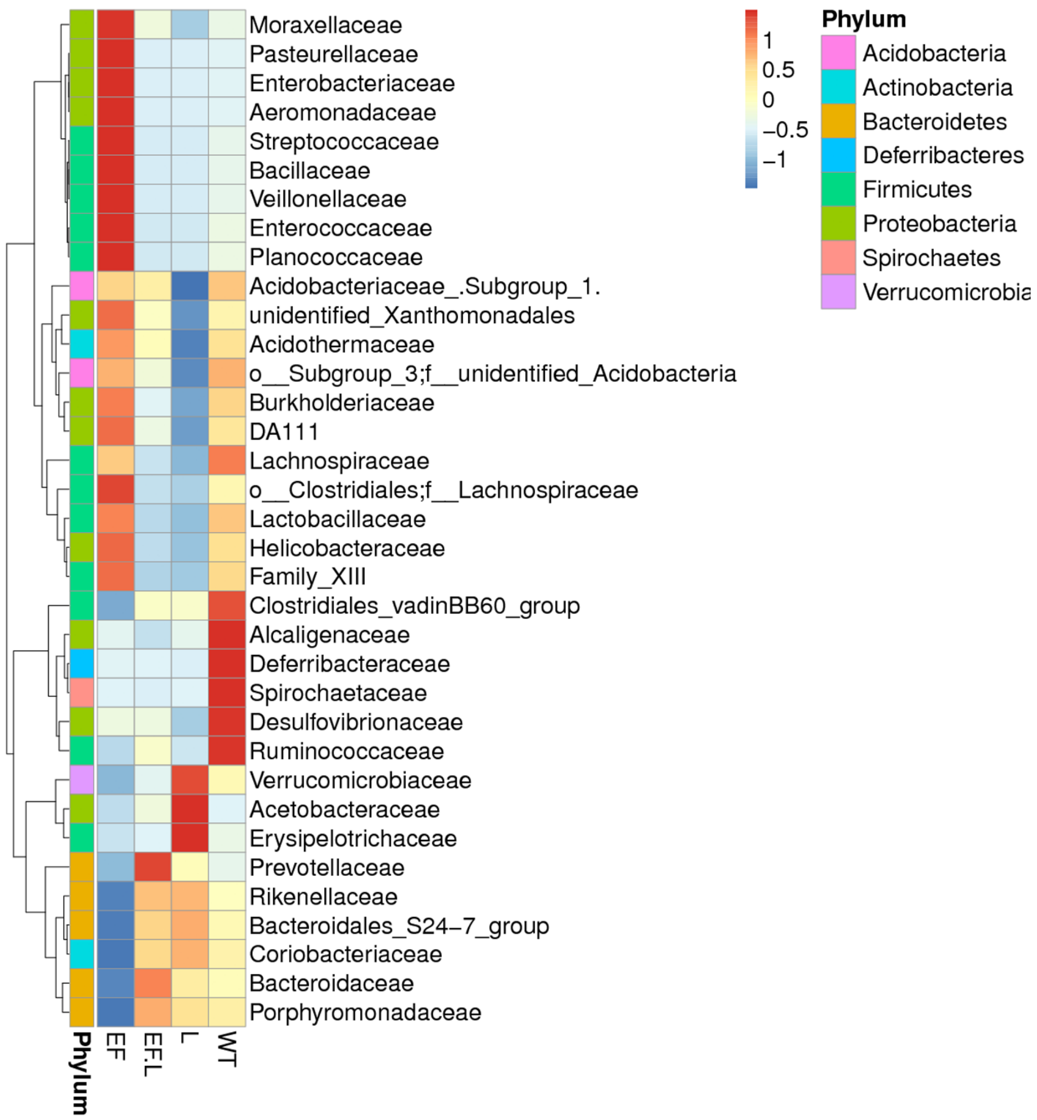

Figure S10. Abundance of intestinal bacteria at the family level.

Table S1. Calcium concentration of LysEF-P10 (0.1 mM) with/without mutation measured by ICP-AES.

	Residue	ICP-AES samples	Ca^{2+} (mM)	Key residues for Ca^{2+} binding[a]
X (1)	D20	D20A	1.1×10^{-4}	●
Y (3)	D22	D22A	0.8×10^{-4}	●
Z (5)	A24	-	-	○
-X (7)	G26	G26A	1.0×10^{-1}	○
-Y (12)	D31	D31A	1.2×10^{-4}	●
		Native LysEF-P10	0.9×10^{-1}	
		buffer	1.0×10^{-4}	

[a] ●: key residue for calcium binding; ○: nonessential residue for calcium binding.

Table S2. Primers used in this study.

Genes names	Primers names	Sequence (from 5' to 3')
LysEF-P10	lys-F	CCG***CTCGAG***ATGGTTAAAGTAAACGATGTA
	lys-R	CG***GGATCC***TTATACCTTAAACTGTGGATG
LysEF-P10C	CHAP-F	CCG***CTCGAG***ATGGTTAAAGTAAACGATGTA
	CHAP-R	CG***GGATCC***TTACGCTGGAGTATCCTTTTCGTA
LysEF-P10C-GFP	CHAPG-F	***GCGGCCTGGTGCCGCGCGGCAGCCATATGCTC***ATGGTTAAAGTAAACGATGT
	CHAPG-R	***CCTCGCCCTTGCTCACGGGGGTTCT***CGCTGGAGTATCCTTTTCGT
LysEF-P10B-GFP	SH3bG-F	***GCGGCCTGGTGCCGCGCGGCAGCCATATGCTC***TACCTTAAACTGTGGATGGG
	SH3bG-R	***CCTCGCCCTTGCTCACGGGGGTTCT***CCGGATCTACCTTAAACTGTGGATGGG
FGFP	FGFP-F	***TCCTTACGAAAAGGATACTCCAGCG***AGAACCCCCGTGAGCAAGGG
	FGFP-R	***GCTTCCTTTCGGGCTTTGTTAGCAGCCGGATC***CTACTTGTACAGCTCGTCCA
GFP	GFP-F	CCG***CTCGAG***AGAACCCCCGTGAGCAAGGG
	GFP-R	CG***GGATCC***CTACTTGTACAGCTCGTCCA
16S rRNA V4 region	515F	GTGCCAGCMGCCGCGGTAA
	806R	GGACTACHVGGGTWTCTAAT

The enzyme sites and homologous arms are underlined.
